# Nutritional Regulation of Ovarian Bioenergetics: Implications for Reproductive Aging and Female Infertility

**DOI:** 10.3390/nu18111773

**Published:** 2026-05-30

**Authors:** Jihyun Kim, Jaewang Lee

**Affiliations:** 1 Research Institute for Pharmaceutical Sciences, College of Pharmacy, Seoul National University, Seoul 08826, Republic of Korea; kimjh763@snu.ac.kr; 2 Division of Biopharmaceutical Sciences, Department of Senior Healthcare, Eulji University, Uijeongbu 11759, Republic of Korea; 3 Department of Biomedical Laboratory Sciences, Eulji University, Seongnam 13135, Republic of Korea

**Keywords:** ovarian metabolism, mitochondrial dysfunction, NAD^+^ metabolism, reproductive aging, nutritional intervention

## Abstract

Ovarian function is critically dependent on tightly coordinated cellular energy metabolism, which governs follicular development, oocyte competence, and reproductive longevity. Increasing evidence indicates that metabolic dysregulation, including mitochondrial dysfunction, oxidative imbalance, and impaired NAD^+^ metabolism, contributes to the pathophysiology of major ovarian disorders such as PCOS, ovarian aging, and DOR. In parallel, emerging studies suggest that nutritional factors influence ovarian function by modulating mitochondrial bioenergetics, redox homeostasis, and nutrient-sensing signaling pathways. This review summarizes current knowledge on the molecular basis of ovarian energy metabolism and its disruption in female reproductive disorders. We further discuss nutritional strategies targeting ovarian bioenergetics, including antioxidants, NAD^+^ precursors, mitochondrial cofactors, and dietary metabolic interventions. In addition, we highlight recent advances in metabolomics, microbiome research, epigenomics, and multi-omics integration that are shaping emerging nutrition-based approaches in reproductive medicine. Collectively, positioning ovarian metabolism at the center of nutritional reproductive research may provide a conceptual framework for understanding metabolic regulation in ovarian function and for guiding future research on reproductive health.

## 1. Introduction

Female reproductive function depends on tightly coordinated cellular energy metabolism [1,2]. The ovary is a metabolically active organ that requires substantial energy to support key processes including folliculogenesis, oocyte maturation, steroidogenesis, and ovulation [3]. Within the ovarian follicle, oocytes and surrounding somatic cells—such as granulosa and cumulus cells—engage in coordinated metabolic interactions that sustain energy production and biosynthetic activity required for successful oocyte development [4]. At the cellular level, ovarian energy metabolism is regulated by interconnected pathways, including mitochondrial oxidative phosphorylation, glycolysis, and lipid metabolism. Oocytes primarily depend on mitochondrial ATP production to maintain meiotic progression and developmental competence, whereas granulosa and cumulus cells supply metabolic substrates through glycolytic and lipid metabolic pathways. These processes are closely linked to redox homeostasis, in which reactive oxygen species (ROS) act as both signaling mediators and drivers of oxidative stress. Disruption of this balance leads to mitochondrial dysfunction, oxidative damage, and impaired follicular development [5,6]. Consequently, alterations in mitochondrial activity and metabolic homeostasis have been associated with reduced oocyte competence, impaired follicular function, and reproductive decline.

Beyond intrinsic cellular metabolism, ovarian function is also influenced by systemic metabolic status and nutritional factors. Accumulating evidence indicates that dietary components, micronutrients, and metabolic intermediates can modulate mitochondrial function, redox balance, and signaling pathways in ovarian cells [7]. Consistent with this framework, ovarian disorders—including diminished ovarian reserve (DOR), polycystic ovary syndrome (PCOS), and endometriosis—are now understood to involve metabolic dysregulation. These conditions are frequently associated with mitochondrial dysfunction, chronic inflammation, and oxidative stress, which collectively impair oocyte quality and follicular function [8,9]. For example, endometriosis is characterized by an inflammatory and oxidative microenvironment that adversely affects ovarian reserve and oocyte competence, highlighting the interplay between systemic and local metabolic disturbances [10]. Although several reviews have addressed individual aspects of reproductive metabolism or nutritional interventions, many have focused on specific pathways or compounds, with limited integration across molecular, metabolic, and systems-level perspectives.

In this review, we aim to address these gaps by (i) providing an integrated overview of ovarian energy metabolism, (ii) critically evaluating its dysregulation across major ovarian disorders, and (iii) highlighting emerging nutritional and multi-omics strategies that may inform targeted metabolic interventions. By linking molecular mechanisms with translational perspectives, this review seeks to provide a conceptual framework for understanding how metabolic regulation can be leveraged to improve reproductive health.

## 2. Energy Metabolism in the Ovary: Molecular and Cellular Basis

### 2.1. NAD^+^ Metabolism in Ovarian Cells

Nicotinamide adenine dinucleotide (NAD^+^) is a central redox cofactor that links glycolysis, the tricarboxylic acid (TCA) cycle, oxidative phosphorylation, and stress-response signaling [11]. Beyond its role in electron transfer, NAD^+^ also serves as a substrate for major NAD^+^-consuming enzymes, including sirtuins, poly(ADP-ribose) polymerases (PARPs), and CD38, thereby coupling cellular energy state to DNA repair, mitochondrial quality control, and metabolic adaptation. In the ovary, these NAD^+^-dependent processes are particularly relevant, as both oocytes and surrounding granulosa and cumulus cells operate under high bioenergetic demand during folliculogenesis, meiotic maturation, and steroidogenesis [12]. Accordingly, disruptions in NAD^+^ availability may adversely affect ovarian function and oocyte developmental competence.

In oocytes, NAD^+^ is closely linked to mitochondrial redox balance and ATP production [13]. Mitochondria provide the ATP required for meiotic progression, spindle assembly, and cytoskeletal dynamics during oocyte maturation. NAD^+^ functions as a key redox carrier in oxidative phosphorylation and the TCA cycle, supporting efficient energy production [14]. Evidence derived primarily from preclinical studies, including in vivo mouse models and in vitro oocyte systems, indicates that reduced NAD^+^ availability is associated with impaired mitochondrial function, increased oxidative stress, and decreased developmental competence in aging oocytes. However, direct clinical evidence in humans remains limited. Recent reviews further suggest that NAD^+^ integrates redox balance with mitochondrial maintenance and chromatin-associated stress responses, supporting its role as an upstream regulator of ovarian aging-related processes [15].

Granulosa and cumulus cells are similarly dependent on NAD^+^-regulated metabolism [16]. These somatic cells support oocyte maturation by supplying metabolic intermediates and maintaining the follicular microenvironment. NAD^+^-dependent enzymes, including sirtuins and PARPs, regulate mitochondrial activity, redox balance, and metabolic adaptation in these cells, thereby contributing to follicular homeostasis and oocyte quality [17]. In women with PCOS, granulosa-cell analyses have reported decreased NAD^+^ levels in association with mitochondrial dysfunction and increased oxidative stress, although these findings are largely based on ex vivo or in vitro analyses. Want et al. demonstrated that granulosa-cell NAD^+^ depletion was accompanied by increased inflammatory cytokine expression, elevated ROS levels, reduced ATP production, and decreased mitochondrial membrane potential [18]. Notably, nicotinamide riboside (NR) restored NAD^+^ levels and improved mitochondrial function in granulosa-like KGN cells, suggesting a functional link between NAD^+^ availability and g granulosa-cell metabolic regulation in a human cell-based model. Collectively, these findings indicated that NAD^+^ metabolism is closely associated with ovarian energy homeostasis, linking mitochondrial bioenergetics, redox balance, and follicular function, although further clinical validation is required.

### 2.2. Mitochondrial Bioenergetics in Folliculogenesis

Mitochondrial bioenergetics plays a central role in follicular development and oocyte maturation. During folliculogenesis, both oocytes and surrounding granulosa cells undergo dynamic metabolic reprogramming to support proliferation, differentiation, and steroidogenesis. Mitochondria generate ATP through oxidative phosphorylation while also contributing to biosynthetic pathways and redox regulation required for follicle growth [19].

Energy production within the ovarian follicle is supported by multiple interconnected pathways, including glycolysis, fatty acid oxidation (FAO), and mitochondrial oxidative phosphorylation [20]. Granulosa cells exhibit relatively high glycolytic activity, converting glucose into intermediates such as pyruvate and lactate. These metabolites are subsequently transported to the oocyte and utilized in mitochondrial oxidative metabolism [4]. In contrast, oocytes rely predominantly on oxidative metabolism, including the TCA cycle and mitochondrial respiration, to generate ATP required for meiotic progression and early developmental competence [21].

FAO provides an additional source of acetyl-CoA and reducing equivalents that support mitochondrial respiration, particularly during later stages of follicular maturation [22]. However, excessive lipid accumulation or dysregulated FOA may contribute to lipotoxicity and oxidative stress, suggesting that tight regulation of these pathways is essential for maintaining mitochondrial integrity and supporting oocyte quality.

### 2.3. Metabolic Coupling Between Oocyte and Granulosa Cells

Within the ovarian follicle, oocytes and surrounding granulosa cells form a metabolically interdependent unit. Because oocytes have limited glycolytic capacity, they rely on somatic cells to supply key metabolic substrates required for energy production. Granulosa and cumulus cells metabolize glucose via glycolysis and transfer intermediates such as pyruvate, lactate, and amino acids to the oocyte through gap junctions and paracrine signaling [23]. This metabolic coupling supports mitochondrial oxidative metabolism and ensures adequate energy supply for oocyte maturation.

In addition to carbohydrate metabolism, granulosa cells contribute to lipid metabolism, redox balance, and nutrient sensing within the follicular microenvironment [24]. Disruption of this metabolic communication have been associated with impaired oocyte maturation and compromised follicular development, although most evidence is derived from preclinical and in vitro studies. Collectively, these findings highlight that ovarian energy metabolism is regulated through complex coordinated interactions between mitochondrial bioenergetics, redox balance, and metabolic coupling between germ cells and somatic follicular cells. Further studies are required to clarify how these interactions translate to human reproductive outcomes.

## 3. Metabolic Dysregulation in Ovarian Disorders

Ovarian function depends on tightly regulated cellular energy metabolism, and growing evidence suggests that metabolic disturbances contribute to the pathogenesis of several ovarian disorders. Although these conditions differ in clinical presentation, they share common features, including mitochondrial dysfunction, oxidative stress, and impaired cellular bioenergetics. These metabolic abnormalities can disrupt follicular development, compromise oocyte competence, and ultimately contribute to infertility [25].

### 3.1. PCOS and Metabolic Dysfunction

PCOS is increasingly recognized not only as an endocrine disorder but also as a state of ovarian metabolic stress characterized by mitochondrial dysfunction, oxidative imbalance, and impaired nutrient handling [26]. These abnormalities have been associated with reduced ATP production, altered glucose metabolism, increased ROS generation, and defective mitochondrial biogenesis in granulosa cells, linking systemic metabolic dysfunction to local follicular impairment [27]. Human granulosa-cell studies provide supporting evidence. In a cohort of 66 women with PCOS and 63 controls, Xie et al. reported significant mitochondrial structural damage in cumulus granulosa cells, including edema, vacuolization, and reduced cristae. Functionally, ATP levels were significantly reduced (1.55 ± 0.78 vs. 0.70 ± 0.35, *p* = 0.006), accompanied by increased ROS, decreased mitochondrial membrane potential, and downregulation of the SIRT1/p-AMPK/PGC-1α signaling axis [28]. These findings suggest impaired mitochondrial biogenesis and energy metabolism in PCOS granulosa cells. In addition, systemic metabolic disturbances such as insulin resistance may further exacerbate ovarian dysfunction by altering glucose utilization, mitochondrial substrate availability, and nutrient-sensing pathways [29]. However, most evidence remains associative, and causal relationships between metabolic dysregulation and reproductive outcomes require further investigation.

### 3.2. Ovarian Aging and Diminished Ovarian Reserve and Poor Ovarian Response

Age-related ovarian decline and DOR are also associated with metabolic dysfunction within the follicular compartment [30]. Increasing evidence suggests that oocyte quality decline in DOR is closely linked to impaired metabolic support from surrounding granulosa and cumulus cells rather than chronological age alone [31]. Ex vivo analyses of patient-derived cumulus granulosa cells have demonstrated abnormal mitochondrial ultrastructure, reduced mitochondrial function, altered mitochondrial dynamics, and decreased mitochondrial mass. These changes are accompanied by downregulation of the SIRT1/p-AMPK/PGC-1α pathway, suggesting impaired mitochondrial biogenesis and quality control in DOR [32]. However, direct clinical implications remain to be established.

Transcriptomic and integrative omics studies further support the association between metabolic dysfunction and reproductive outcomes. In a cohort of 99 cumulus-cell samples, Zhang et al. reported that DOR—independent of age—was associated with poorer assisted reproductive technology (ART) outcomes, whereas advanced age with preserved ovarian reserve did not show comparable deterioration. At the molecular level, these outcomes were associated with enrichment of oxygen metabolism-related pathways, highlighting the importance of bioenergetic regulation in somatic follicular cells [33]. Complementary integrative analyses have provided additional insights. Yu et al. demonstrated that granulosa-cell transcriptomics and follicular-fluid metabolomics in DOR are associated with alterations in cholesterol metabolism, fatty acid β-oxidation, and extracellular matrix organization. These changes were accompanied by increased apoptosis, reduced proliferation, and downregulation of antioxidant-related genes such as GPX4 and SLC7A11, as well as ferroptosis-like mitochondrial morphology [34]. These findings suggest that metabolic dysregulation in DOR involves not only impaired energy production but also increased susceptibility to oxidative and lipid peroxidation-related cell damage. Overall, current evidence indicates that metabolic alterations in follicular somatic cells are associated with reduced oocyte competence and poorer ART outcomes. However, these findings are largely derived from observational and molecular profiling studies, and causal relationships remain to be established.

### 3.3. Endometriosis-Associated Ovarian Dysfunction and Chemotherapy-Induced Ovarian Damage

In addition to PCOS and ovarian aging, metabolic dysregulation has also been implicated in other conditions that impair ovarian function, including endometriosis-associated ovarian dysfunction and chemotherapy-induced ovarian damage. Endometriosis is increasingly recognized as a condition characterized by chronic inflammation, oxidative stress, and mitochondrial dysfunction within the ovarian microenvironment [35]. Clinical and experimental studies have reported increased oxidative stress and impaired mitochondrial activity in granulosa cells and follicular fluid from women with endometriosis, which may adversely affect oocyte quality and follicular development [36].

Chemotherapy-induced ovarian damage can similarity be viewed as a disorder of the ovarian microenvironment and its metabolic integrity. Chemotherapeutic agents induce mitochondrial injury, excessive ROS production, and disruption of cellular energy metabolism [37]. Guo et al. reported that chemotherapy perturbs the ovarian niche at multiple levels, including stromal fibrosis, vascular injury, immune imbalance, oxidative stress, and accumulation of senescent cells, ultimately reducing both follicle quantity and quality [38]. These findings suggest that ovarian failure following chemotherapy reflects not only direct oocyte damage but also broader metabolic and microenvironmental disruption. However, most available evidence is derived from experimental and observational studies, and the extent to which these mechanisms translate to clinical fertility outcomes remains to be clarified.

## 4. Nutritional Strategies Targeting Ovarian Energy Metabolism

Metabolic dysfunction is increasingly recognized as a central feature of ovarian disorders, including PCOS, ovarian aging, and DOR. Given the critical role of mitochondrial bioenergetics and redox homeostasis in follicular development, nutritional interventions that modulate ovarian metabolism have gained growing interest. Various nutrients and dietary strategies have been reported to influence mitochondrial function, NAD^+^ metabolism, oxidative stress responses, and nutrient-sensing signaling pathways. These approaches represent mechanistically plausible strategies for modulating ovarian metabolism; however, evidence for clinically meaningful reproductive outcomes remains limited.

### 4.1. Antioxidants and Redox Regulation

Oxidative stress is particularly detrimental in the ovary, where mitochondrial ATP production is required for spindle assembly, chromosome segregation, and early embryonic competence. Excessive ROS can damage mitochondrial membranes, impair mtDNA integrity, and disrupt metabolic cooperation between granulosa cells and the oocyte. Recent studies therefore position mitochondrial redox imbalance as a central feature of ovarian aging and infertility rather than a secondary by-product [39,40].

Resveratrol functions not only as an antioxidant but also as a redox-sensitive metabolic modulator. It has been shown to activate SIRT1 and AMP-activated protein kinase (AMPK), thereby promoting mitochondrial biogenesis and oxidative metabolism [41,42]. A recent systematic review including both 9 clinical studies and 15 in vitro studies reported potential improvements in ovarian function, mitochondrial activity, and oocyte quality; however, clinical evidence remains limited and heterogeneous, and optimal dosing, treatment duration, and safety considerations remain unresolved [43]. Melatonin has been widely studied for its roles in redox regulation and mitochondrial function. Physiologically, it contributes to antioxidant defense and circadian regulation within ovarian cells. Experimental studies have demonstrated that melatonin can enhance mitochondrial function and modulate autophagy-related pathways in oocytes, while in vitro studies in human granulosa cells suggest involvement of defined signaling pathways, including SIRT1 and PDK1/Akt [44]. However, most evidence remains preclinical, and translation to clinical reproductive outcomes remains uncertain.

Flavonoid-based compounds, including quercetin and curcumin, represent a broader class of metabolic antioxidants with anti-inflammatory and insulin-sensitizing properties. Experimental studies indicate that these compounds can modulate oxidative stress, mitochondrial function, and metabolic signaling pathways, while clinical studies have primarily reported improvements in metabolic and endocrine parameters, particularly in PCOS [45,46]. These compounds are best integrated as multi-target metabolic modulators rather than direct fertility-enhancing agents.

Beyond these compound, additional nutritional factors—including phytoestrogens, dietary-derived growth factors (e.g., IGF-1), omega-3 fatty acids, and vitamins—have been implicated in ovarian metabolic regulation [47,48,49,50]. However, their effects are often context-dependent, and current evidence for direct improvements in key reproductive endpoints, such as implantation or live birth, remains limited and inconclusive.

Overall, while various nutritional compounds show promise in modulating ovarian metabolism and early reproductive parameters, robust clinical evidence demonstrating improvements in definitive reproductive outcomes remains limited. Clinical studies evaluating antioxidant-based nutraceutical interventions are summarized in Table 1.

### 4.2. NAD^+^-Boosting Nutrients and NAD-Dependent Metabolic Signaling

NAD^+^-boosting strategies represent one of the most mechanistically grounded nutritional approaches for modulating ovarian bioenergetics. Unlike general antioxidants, NAD^+^ precursors target a central metabolic node that regulates redox balance, mitochondrial respiration, DNA repair, and NAD^+^-dependent signaling pathways, including sirtuins and PARPs. This positions NAD^+^ metabolism as a potential integrator of cellular energy status and stress responses in ovarian cells. Among these compounds, NMN has been extensively studied in preclinical models. In vivo studies in aged mice have shown that NMN supplementation can restore oocyte NAD^+^ levels and is associated with improvements in ovulation, meiotic competence, and early developmental parameters. Mechanistically, these effects have been linked to enhanced mitochondrial function, reduced oxidative stress, and decreased apoptosis, based on transcriptomic and functional analyses [61,62]. However, these findings are derived from animal models, and their relevance to human reproductive outcomes remains to be established.

Nicotinamide riboside (NR) is another NAD^+^ precursor with emerging relevance in ovarian metabolic regulation. In a dehydroepiandrosterone (DHEA)-induced PCOS mouse model, NR supplementation has been reported to restore ovarian NAD^+^ levels, improve estrous cyclicity, and enhance ovulatory function [63]. At the cellular level, NR treatment was associated with improved mitochondrial function, reduced oxidative stress, and improved oocyte-related parameters, along with modulation of sirtuin signaling and suppression of fibrosis-related pathways such as TGF-β/SMAD3. These findings suggest that NR may influence ovarian function through combined effects on mitochondrial metabolism and stromal remodeling. Nevertheless, current evidence is limited to preclinical models, and clinical validation is lacking. Evidence supporting clinical efficacy of NAD^+^-targeting interventions in reproductive outcomes remains limited. The available clinical studies are summarized in Table 2.

### 4.3. Nutrients Regulating Mitochondrial Bioenergetics

Mitochondrial bioenergetics depends on coordinated activity of the electron transport chain and multiple metabolic pathways supplying reducing equivalents. Nutrients that support mitochondrial function may therefore contribute to maintaining ovarian metabolic homeostasis.

Coenzyme Q10 (CoQ10) functions as both an electron carrier within the mitochondrial respiratory chain and a redox-active molecule. Clinical studies have evaluated CoQ10 supplementation in women with DOR or poor ovarian response using regimens ranging from 600 mg/day for 2 months to 1200 mg/day for 12 weeks. While some studies report improvements in oocyte or embryo-related parameters, findings remain inconsistent across trials [68,69].

L-carnitine plays a key role in FAO by facilitating the transport of long-chain fatty acids into mitochondria. Experimental studies have shown that acyl-carnitines can improve mitochondrial function under oxidative stress conditions in oocytes, supporting the concept that carnitine may enhance metabolic flexibility [70]. Clinical evidence, including a recent meta-analysis, suggests potential benefits of L-carnitine in women with PCOS; however, larger and longer randomized controlled trials are required to confirm these effects [71].

α-Lipoic acid functions as a mitochondrial cofactor and redox-active compound involved in oxidative enzyme complexes. Clinical studies have primarily examined its role in improving insulin sensitivity and metabolic parameters in PCOS. These effects may indirectly influence ovarian function by improving systemic and follicular metabolic environments [72]. However, direct evidence linking α-lipoic acid to reproductive outcomes remains limited.

### 4.4. Dietary Metabolic Interventions

Dietary interventions modulate ovarian metabolism primarily through systemic nutrient sensing and metabolic regulation. Unlike single-compound supplementation, approaches such as caloric restriction (CR), macronutrient composition, and dietary patterns influence key signaling pathways—including AMPK, mTOR, and sirtuins—thereby affecting mitochondrial function, inflammation, and substrate utilization within the ovarian microenvironment [73,74].

CR represents a well-characterized metabolic intervention. In a recent mouse study, Prosczek et al. demonstrated that long-term CR (3–11 months) improved insulin sensitivity and preserved the primordial follicle pool, suggesting that both duration and timing of metabolic intervention influence ovarian aging [75]. However, evidence in humans remains limited, and the applicability of CR to reproductive outcomes requires further investigation.

Dietary patterns have also been associated with reproductive metabolic outcomes. The Mediterranean diet, characterized by a high intake of fruits, vegetables, whole grains, and unsaturated fats, has been linked to improved metabolic health and fertility-related parameters in observational studies [76]. In women with PCOS, low-glycemic and insulin-sensitizing dietary approaches have been associated with improvements in metabolic and endocrine profiles, which may indirectly influence ovarian function [77]. However, these findings are largely associative and do not consistently demonstrate direct effects on reproductive endpoints.

Beyond overall dietary patterns, specific nutritional components may contribute to ovarian metabolic regulation. Protein intake and amino acid metabolism can influence insulin signaling and mTOR activity [78], while dietary lipids, including omega-3 fatty acids, may modulate inflammatory and mitochondrial pathways [79]. In addition, emerging evidence suggests that gut microbiome-mediated nutrient metabolism may influence systemic and ovarian metabolic homeostasis, although mechanistic and clinical data remain limited [80].

Collectively, these findings suggest that dietary interventions may influence ovarian metabolism through integrated systemic and local mechanisms. However, current evidence is heterogeneous, and further studies are required to define optimal dietary strategies and their impact on clinically meaningful reproductive outcomes.

### 4.5. Safety Considerations and Translational Limitations

Nutritional interventions targeting ovarian metabolism—including NAD^+^ boosters, antioxidants, and mitochondrial cofactors—require careful consideration of their safety and translational applicability. Several factors may influence their clinical relevance, including context-dependent biological effects, pharmacokinetic limitations, and heterogeneity in study design.

NAD^+^-boosting strategies such as NMN and NR have shown beneficial effects in preclinical models; however, NAD^+^ is a central regulator of cellular metabolism, DNA repair, and survival pathways. As a result, systemic modulation of NAD^+^ may have context-dependent effects, particularly in conditions involving altered proliferative signaling, such as cancer [81,82].

Antioxidant compounds may also exert stage- and context-dependent effects. For example, while resveratrol has been associated with improved mitochondrial function, it has been reported to inhibit decidualization in human endometrial stromal cells, suggesting potential effects on endometrial receptivity depending on timing and physiological context [83]. Similarly, melatonin, although widely used for its antioxidant properties, is a neuroendocrine regulator that may influence circadian and hormonal signaling, particularly at supraphysiological doses [84].

Pharmacokinetic limitations further complicate translation. Many bioactive compounds, including curcumin and flavonoids, exhibit low oral bioavailability, raising uncertainty regarding their effective concentrations in ovarian tissue [85]. In addition, clinical studies are often heterogeneous in terms of formulation, dosage, treatment duration, and patient characteristics and frequently rely on surrogate endpoints rather than definitive reproductive outcomes such as live birth [86].

Collectively, these considerations indicate that while nutritional modulation of ovarian metabolism is mechanistically promising, its clinical application requires cautious interpretation and further validation.

## 5. Emerging Omics Approaches for Nutritional Reproductive Medicine

### 5.1. Metabolomics Reveals Nutrient-Dependent Metabolic Rewiring

Recent metabolomic and lipidomic studies indicate that the follicular microenvironment is dynamically remodeled in ovarian disorders. In women with PCOS, alterations in follicular-fluid composition—including lipid and metabolic signatures—have been consistently reported, suggesting that follicular dysfunction involves substantial metabolic dysregulation beyond endocrine imbalance. For example, He et al. demonstrated that follicular-fluid lipidomics revealed abnormalities in glycerophospholipid and sphingomyelin metabolism, linking systemic metabolic dysfunction to the biochemical environment surrounding the oocyte [87]. Similarly, metabolomic analyses of ovarian aging have identified disruptions in pathways related to lipid metabolism, folate metabolism, and mitochondrial cofactors. Zeng et al. reported that both physiological and pathological ovarian aging share disturbances in lipid, folate, and ubiquinone metabolism, while distinct alterations involve vitamin and retinol pathways, highlighting the metabolic heterogeneity of ovarian aging [88]. These findings suggest that ovarian dysfunction involves multiple nutrient-sensitive pathways beyond single-axis mechanisms.

Importantly, these observations provide a framework for identifying nutrient-responsive metabolic pathways that may inform targeted nutritional strategies. For instance, dysregulated lipid metabolism may indicate potential roles for dietary fatty acid composition, while alterations in folate and mitochondrial metabolism may highlight additional nutrient-dependent pathways for future investigation [89]. However, most metabolomic studies remain cross-sectional and are limited by small sample sizes, batch effects, and variability in analytical platforms. These limitations restrict causal interpretation and highlight the need for longitudinal and interventional validation.

### 5.2. The Microbiome–Ovary Axis

The microbiome–ovary axis has emerged as a potential interface linking diet, metabolism, and reproductive function. The gut microbiome contributes to estrogen metabolism through the estrobolome, thereby influencing systemic estrogen availability and potentially contributing to estrogen-dependent conditions. Pai et al. reported that dysregulation of the estrobolome is associated with disorders such as endometriosis, supporting a link between microbial metabolism and ovarian function [90]. In PCOS, gut dysbiosis has been associated with insulin resistance, chronic inflammation, and altered metabolic responses, suggesting that microbiome-mediated mechanisms may contribute to reproductive dysfunction [91,92]. These findings support the concept that dietary or microbiome-targeted interventions could influence ovarian metabolism; however, current evidence remains largely associative.

Dietary modulation represents a key determinant of microbiome composition. Interventions such as increased fiber intake, prebiotic supplementation, and probiotic administration have been proposed as strategies to restore microbial balance and improve metabolic homeostasis. In this context, microbiome profiling may facilitate identification of patient subgroups that are more likely to respond to specific interventions [93,94].

Nevertheless, microbiome composition is highly variable across individuals and influenced by environmental and methodological factors, limiting reproducibility. Moreover, causal relationships between microbiome alterations and reproductive outcomes remain insufficiently established. Therefore, while microbiome-targeted strategies are conceptually promising, their clinical efficacy requires further validation.

### 5.3. Epigenomic Responses to Nutritional Signals

Nutritional and metabolic signals are increasingly recognized as regulators of epigenetic landscapes. Metabolic cofactors, including NAD^+^, directly influence the activity of epigenetic enzymes such as sirtuins and poly(ADP-ribose) polymerases (PARPs), thereby linking cellular energy status to chromatin remodeling and gene expression. Through these mechanisms, nutritional status may influence gene expression programs involved in follicular development, ovarian aging, and reproductive competence. Nutrigenomic studies further suggest that dietary components can modulate gene expression via DNA methylation, histone modification, and non-coding RNA regulation. For example, Singh et al. proposed an integrative nutrigenomics framework for PCOS, highlighting that metabolic and inflammatory pathways implicated in the disorder may be responsive to dietary modulation through epigenetic mechanism [95]. However, current evidence remains limited by several factors, including restricted access to relevant ovarian tissues, cellular heterogeneity, and a lack of longitudinal data linking epigenetic changes to reproductive outcomes. In addition, distinguishing causal epigenetic regulation from secondary metabolic effects remains challenging. Therefore, while epigenomic insights provide a mechanistic basis for potential nutritional modulation, their clinical relevance remains to be established.

### 5.4. Multi-Omics Integration and Precision Reproductive Nutrition

Integration of metabolomics, microbiome profiling, epigenomics, and genomics is increasingly enabling a systems-level understanding of ovarian metabolism and reproductive function. Multi-omics approaches facilitate the identification of molecular and metabolic signatures associated with ovarian aging, infertility, and variability in treatment response [96]. From a translational perspective, these approaches may support the development of stratified nutritional strategies. By integrating metabolic, microbial, and epigenetic profiles, it may become possible to define ovarian bioenergetic phenotypes and identify patient subgroups with distinct metabolic characteristics. Such stratification could inform individualized dietary or nutraceutical interventions tailored to specific metabolic and inflammatory profiles [97,98].

However, several challenges limit clinical translation. These include batch effects across platforms, data integration and harmonization issues, high dimensionality relative to sample size, and a lack of standardized analytical pipelines. Moreover, longitudinal multi-omics datasets linking molecular signatures to clinically meaningful reproductive outcomes remain limited. Accordingly, while multi-omics approaches offer valuable insights into ovarian metabolic regulation, their application in clinical reproductive medicine remains at an early stage and requires further validation.

## 6. Therapeutic Perspectives and Future Directions

Advances in metabolic and nutritional research have underscored the importance of energy metabolism in ovarian function and reproductive aging. However, despite increasing mechanistic insight, translation of these findings into clinically applicable strategies remains limited. Addressing key knowledge gaps across mechanistic, epidemiological, experimental, and translational domains will be essential for advancing this field.

### 6.1. Defining Molecular Mechanisms Linking Nutrition and Ovarian Metabolism

A major challenge is the incomplete characterization of molecular pathways linking nutritional status to ovarian metabolic regulation. While mitochondrial function, redox balance, and NAD^+^ metabolism have been identified as key components, upstream nutrient-sensing mechanisms and downstream transcriptional responses remain insufficiently defined. Future studies integrating single-cell multi-omics, metabolic flux analyses, and advanced in vitro ovarian models may help clarify how nutritional cues influence the follicular microenvironment.

### 6.2. Establishing Long-Term Population-Level Evidence

At the population level, the relationship between dietary patterns and ovarian aging remains poorly characterized. Most available studies are cross-sectional or focus on short-term reproductive outcomes. Longitudinal cohort studies incorporating detailed dietary data, metabolic biomarkers, and ovarian reserve metrics are required to determine whether sustained nutritional patterns influence reproductive lifespan.

### 6.3. Improving Experimental Models for Nutritional Reproductive Biology

The development of physiologically relevant experimental systems represents another priority. While animal models and in vitro models have provided valuable insights, they often fail to capture the complexity of human ovarian metabolism. Emerging platform, including organoid-based systems, co-culture models, multi-omics integration, may provide more accurate tools for investigating nutrient–metabolism interactions in the ovary.

### 6.4. Translating Metabolic Nutrition into Clinical Reproductive Practice

**Figure 1:**
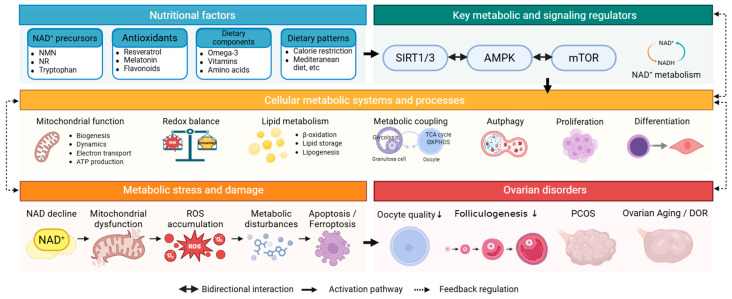
Hierarchical and interconnected regulation of ovarian metabolic function by nutritional factors. Disruption of these metabolic networks leads to metabolic stress and damage, characterized by NAD^+^ decline, mitochondrial dysfunction, ROS accumulation, metabolic disturbances, and cell death pathways, including apoptosis and ferroptosis. These alterations are associated with the development of ovarian disorders, including polycystic ovary syndrome (PCOS), diminished ovarian reserve (DOR), and ovarian aging. Arrows indicate hierarchical regulation, bidirectional interactions, and feedback mechanisms between metabolic regulators, cellular systems, and pathological outcomes.

Clinical integration of nutritional strategies into reproductive medicine requires robust interventional evidence. Modulation of mitochondrial function, oxidative stress, and metabolic homeostasis may complement existing infertility treatments; however, standardized protocols and patient stratification strategies remain lacking. Future clinical trials incorporating metabolic phenotyping and stratified nutrition approaches will be necessary to evaluate their relevance for reproductive outcomes (Figure 1).

## 7. Conclusions

Ovarian function is closely dependent on tightly coordinated cellular energy metabolism, which governs follicular development, oocyte competence, and reproductive longevity. Current evidence—derived largely from in vitro studies, animal models, and limited clinical investigations—suggests that nutritional factors may influence ovarian function through interconnected mechanisms involving mitochondrial bioenergetics, redox regulation, NAD^+^ metabolism, and nutrient-sensing signaling pathways. Disruption of these metabolic networks has been associated with major ovarian disorders, including PCOS, ovarian aging, and diminished ovarian reserve (DOR).

However, the majority of available data remain preclinical or observational, and substantial heterogeneity exists across study design, model systems, and intervention strategies. In addition, differences in dosage, treatment duration, and study populations further limit direct comparison across studies. Evidence linking nutritional interventions to clinically meaningful reproductive outcomes, such as implantation and live birth, remains limited.

Accordingly, while targeting ovarian bioenergetics represents a promising conceptual approach, its clinical application requires cautious interpretation. Future research should focus on integrating mechanistic insights with well-designed longitudinal and interventional studies to establish causality and therapeutic relevance.

Advances in multi-omics technologies offer opportunities to better characterize metabolic heterogeneity and may support the development of more targeted and individualized strategies. Ultimately, improving our understanding of ovarian metabolic regulation may contribute to the development of evidence-based approaches for preserving reproductive function and improving fertility outcomes. Importantly, distinguishing mechanistic effects from clinically meaningful reproductive outcomes remains a key challenge in the field.

## Figures and Tables

**Table 1:** Clinical studies of antioxidant-based interventions for ovarian functions and reproductive outcomes.

Compound	Clinical Population	Study Design	Intervention	Main Findings	Interpretation for Ovarian Function	Ref.
Melatonin	Women with DOR undergoing ART	Double-blind randomized clinical trial; 80 enrolled, 66 analyzed	3 mg/day from day 5 of the cycle preceding gonadotropin stimulation until oocyte pickup	Higher serum estradiol on trigger day; higher proportion of women with mature MII oocytes and grade 1/1–2 embryos; no clear difference in other ART outcomes	May improve oocyte maturity and embryo quality, but evidence for pregnancy/live birth benefit remains limited	[51]
	PCOS patients undergoing IUI	Double-blind randomized clinical trial; 198 women	3 mg/day from menstrual day 3 until hCG administration	Chemical pregnancy rate was higher with melatonin (~32% vs. 18%); endometrial thickness also improved	Suggests potential benefit in PCOS-related subfertility, though endpoint was chemical pregnancy rather than live birth	[52]
	PCOS women undergoing IVF	Clinical trial; 320 women randomized	Melatonin plus metformin vs. metformin-based control; melatonin 3 mg with metformin 500 mg, three times daily	Higher MII oocyte proportion, higher top-quality embryo rate, and higher odds of clinical pregnancy in the intervention arm	Supports adjunctive use in PCOS IVF, but co-treatment with metformin makes melatonin-specific attribution less clean	[53]
Resveratrol	Women > 35 years with good ovarian reserve undergoing IVF	Exploratory randomized placebo-controlled trial; 37 cases and 33 controls	150 mg/day for 3 months before ovarian stimulation	Evaluated ovarian responsiveness in advanced reproductive-age women (follicle output rate, follicle-to-oocyte index improved)	Human fertility evidence is still preliminary; useful as supportive but not definitive clinical evidence	[54]
	PCOS women undergoing ART	Randomized, triple-blind, placebo-controlled clinical trial; 56 patients	800 mg/day for 60 days before oocyte collection	Improved mitochondrial biogenesis-related markers in granulosa cells, mtDNA copy number, ATP content, and oocyte maturity/embryo quality indices	Stronger mechanistic human evidence that resveratrol may support granulosa-cell mitochondrial function and early ART parameters	[55]
Curcumin	Women with PCOS	Randomized double-blind placebo-controlled trial; 72 enrolled, 67 analyzed	500 mg three times daily for 12 weeks	Improved glucose-related indices and insulin resistance; trial focused on metabolic and androgen outcomes rather than direct fertility endpoints	Clinical signal is mainly metabolic, not direct ovarian reserve or ART efficacy	[56]
	Women with PCOS	Systematic review/meta-analysis of clinical studies	Various formulations/doses	Beneficial effects on inflammation, body weight, glucose and lipid metabolism; safety acceptable, but larger definitive trials needed	Better positioned as a metabolic adjunct in PCOS rather than proven fertility enhancer	[57,58]
Quercetin	Women with PCOS	Randomized placebo-controlled double-blind clinical trial; 84 women	1 g/day for 12 weeks	Increased adiponectin/HMW adiponectin; reduced testosterone, LH, and HOMA-IR	Human evidence supports metabolic and endocrine improvement in PCOS, but not yet robust direct evidence for ovarian reserve restoration or ART success	[59]
	Infertile women undergoing ART	Double-blind randomized clinical trial; 72 women	50 mg/day from menstrual onset until ovulation	Reduced LH and inflammatory cytokines (IL-6); improved oocyte quality, embryo grade, and pregnancy rate	Best described as promising but still early-stage clinical evidence	[60]
Vitamin D	Women undergoing IVF	Systematic review/meta-analysis; 2700+ women (pooled)		Higher clinical pregnancy rates observed in vitamin D-sufficient women; no consistent improvement in live birth rates across studies	May be associated with improved IVF outcomes, but causality remains unclear due to heterogeneity and confounding factors	Vitamin D and in vitro fertilization: a systematic review
Omega-3 fatty acids	Women with PCOS	Randomized double-blind placebo-controlled; 60 women	Omega-3 supplementation (1000 mg/day) for 12 weeks	Improved insulin resistance, decreased inflammatory markers, and improved lipid profile; no direct fertility endpoint assessed	May improve ovarian environment indirectly through metabolic and anti-inflammatory effects, but direct impact on fertility outcomes remains unclear	The Effects of Flaxseed Oil Omega-3 Fatty Acids Supplementation on Metabolic Status of Patients with Polycystic Ovary Syndrome: A Randomized, Double-Blind, Placebo-Controlled Tria
Vitamin D + Omega-3	Women with PCOS	Randomized double-blind placebo-controlled; 60 women	Vitamin D (50,000 IU every 2 weeks) plus omega-3 (1000 mg twice daily) for 12 weeks	Improved insulin resistance, reduced serum testosterone levels, decreased inflammatory markers, and modulated expression of metabolic-related genes; no direct reproductive outcomes assessed	May influence metabolic or embryological parameters, but evidence for live birth outcomes remains limited	The influences of vitamin D and omega-3 co-supplementation on clinical, metabolic and genetic parameters in women with polycystic ovary syndrome
Phytoestrogens (isoflavones)	Women with PCOS	Randomized controlled trial; 70 women	Soy isoflavone supplementation (~50 mg/day) for 12 weeks	Reduced testosterone levels, improved insulin resistance and lipid metabolism; reproductive outcomes not evaluated	May modulate endocrine and metabolic parameters, but effects on fertility are uncertain and potentially dose-dependent	The Effects of Soy Isoflavones on Metabolic Status of Patients With Polycystic Ovary Syndrome

DOR, diminished ovarian reserve; PCOS, polycystic ovary syndrome; ART, assisted reproductive technologies; IUI, intrauterine insemination; IVF, in vitro fertilization.

**Table 2:** Clinical evidence regarding NAD^+^-related and sirtuin-targeting metabolic interventions in ovarian health.

Compound/Strategy	Clinical Population	Study Design	Intervention	Main Findings	Interpretation	Ref.
NADH	Immature human oocytes discarded from controlled ovarian hyperstimulation cycles	Human oocyte-based pilot study in IVM setting	IVM medium supplemented with NADH (optimal concentration identified as 10^−6^ M)	Increased maturation rate, blastocyst rate, ATP, mitochondrial membrane potential, and glutathione; reduced ROS	This is human translational evidence, but it is not an oral/systemic clinical supplementation trial in women	[64]
NR/NMN/oral NAD^+^ precursors	Women with infertility, DOR, POI, or ovarian aging	data	data	No robust direct clinical trial in women identified for oral NR/NMN/NAD^+^ precursor supplementation with ovarian reserve or ART endpoints	Current evidence remains largely preclinical/animal-based; should be described as a major translational gap	[12,65]
Sirtuin-targeting interventions	Women with reproductive disorders	data	data	Human data are mostly indirect, mechanistic, or observational; no established ovarian-targeted RCT demonstrating improved fertility endpoints via direct sirtuin modulation	At present, sirtuins are better framed as mechanistic mediators than clinically validated ovarian therapeutics	[66,67]

DOR, diminished ovarian reserve; POI, primary ovarian insufficiency; PCOS, polycystic ovary syndrome; RCT, randomized clinical trials.

## Data Availability

No new data were created or analyzed in this study. Data sharing is not applicable to this article.

## Abbreviations

The following abbreviations are used in this manuscript:

ATP	Multidisciplinary Digital Publishing Institute
TCA	Tricarboxylic Acid
NAD	Nicotinamide Adenine Dinucleotide
PARPs	Poly(ADP-ribose) Polymerases
NR	Nicotinamide Riboside
PCOS	Polycystic Ovarian Syndrome
FAO	Fatty Acid Oxidation
ROS	Reactive Oxygen Species
DOR	Diminished Ovarian Reserve
ARTs	Assisted Reproductive Technologies
CR	Calorie Restriction
